# Mortality in Patients with 22q11.2 Rearrangements

**DOI:** 10.3390/genes15091146

**Published:** 2024-08-30

**Authors:** Melisa Cilio Arroyuelo, Jair Tenorio-Castano, Luis Fernández García-Moya, Alejandro Parra, Mario Cazalla, Natalia Gallego, Lucía Miranda, María Ángeles Mori, Luis García-Gueretta, Carlos Labrandero, Elena Mansilla, Emi Rikeros, Fe García-Santiago, Isabel Vallcorba, Pedro Arias, Cristina Silván, Lucia Deiros Bronte, Julián Nevado, Pablo Lapunzina

**Affiliations:** 1 Institute of Medical and Molecular Genetics, Hospital Universitario La Paz, INGEMM-IdIPAZ, 28046 Madrid, Spainjairantonio.tenorio@gmail.com (J.T.-C.); lfernandezg@salud.madrid.org (L.F.G.-M.); alejandro.parra.ingemm@gmail.com (A.P.); mario.cazalla16@gmail.com (M.C.); nataliagallegozazo@gmail.com (N.G.); luciamiranda.ingemm@gmail.com (L.M.); mangeles.mori@salud.madrid.org (M.Á.M.); elena.mansilla@salud.madrid.org (E.M.); emikarina.rikeros@salud.madrid.org (E.R.); feamalia.garcia@salud.madrid.org (F.G.-S.); isabel.vallcorba@salud.madrid.org (I.V.); palajara@gmail.com (P.A.); cristina_sf8@hotmail.com (C.S.); jnevadobl@gmail.com (J.N.); 2 Centro de Investigación Biomédica en Red de Enfermedades Raras, CIBERER, 28029 Madrid, Spain; 3 European Reference Network, ITHACA, 1070 Brussels, Belgium; 4 Department of Pediatric Cardiology, Hospital Universitario La Paz, 28046 Madrid, Spain; lggueretasilva@salud.madrid.org (L.G.-G.); carlos.labrandero@salud.madrid.org (C.L.); lucia.deiros@salud.madrid.org (L.D.B.)

**Keywords:** 22q11.2 microdeletion syndrome, 22q11.2 microduplication syndrome, Cat Eye Syndrome, Emmanuel syndrome, mortality, congenital heart defects, 22q11.2 rearrangement, arrayCGH, MLPA, FISH

## Abstract

The 22q11.2 region is highly susceptible to genomic rearrangements leading to multiple genomic disorders, including 22q11.2 microdeletion syndrome (22q11.2 DS) (MIM# 188400), 22q11.2 microduplication syndrome (MIM# 608363), supernumerary der(22)t(11;22) syndrome (also known as Emanuel Syndrome; MIM# 609029), and Cat Eye Syndrome (MIM# 115470). In this study, we present data on causes of mortality, average age of death, and the existing associated risk factors in patients with 22q11.2 rearrangements. Our cohort included 223 patients (120 males and 103 females) with confirmed diagnoses of 22q11.2 rearrangements diagnosed through molecular techniques (FISH, MLPA, and CMA). Relatives from patients who have been molecularly confirmed with 22q11.2 rearrangements have also been added to the study, regardless of the presence or absence of symptoms. Of these 223 individuals, 21 (9.4%) died. Deceased patients’ rearrangements include 19 microdeletions, 1 microduplication, and 1 patient with a marker chromosome. The median age of death was 3 months and 18 days (ranging from 3 days to 34 years). There were 17 patients who died at pediatric age (80.95%), 3 died at adult age (14.28%), and for 1 of whom, the age of death is unknown (4.76%). Eighteen patients were White Mediterranean (European non-Finnish) (85.71%) whereas three were Amerindian (South American) (14.28%). Mortality from cardiac causes accounted for 71.42%. The second most frequent cause of death was sepsis in two patients (9.52%). One patient died from respiratory failure (4.76%) and one from renal failure (4.76%). Information regarding the cause of death was not available in two patients (9.52%). Most patients who died were diagnosed within the first week of life, the majority on the first day. This study adds additional information on mortality in one of the largest cohorts of patients with 22q11.2 rearrangements in more than 30 years of follow-up.

## 1. Introduction

The 22q11.2 genomic region is highly susceptible to rearrangements leading to multiple genomic disorders, including 22q11.2 microdeletion syndrome (22q11.2 DS; MIM# 188400), 22q11.2 microduplication syndrome (MIM# 608363), supernumerary der(22)t(11;22) syndrome, also known as Emanuel Syndrome (ES; MIM# 609029), and Cat Eye Syndrome (CES; MIM# 115470) [1]. From these, the most frequent is the 22q11.2 DS, which is the most common chromosomal microdeletion disorder in humans caused by the loss of the q11.2 region of chromosome 22. The estimated incidence is approximately ~1/2150 live births, affecting both sexes equally [2]. Most patients (90–95%) have de novo rearrangements [3]; the remaining percentage corresponds to hereditary cases, in which there is a 50% risk of recurrence with 100% penetrance and variable expressivity [4]. Clinical features are highly variable and heterogeneous, including dysmorphic features, immunodeficiency (due to thymus aplasia or hypoplasia), endocrinologic disorders such as hypocalcemia caused by hypoplasia of the parathyroid glands, palatal anomalies, cardiac abnormalities, which are notably the most prevalent, neuropsychiatric conditions, developmental delay, and intellectual disability. This variability may contribute to a delayed diagnosis, with numerous patients taking years to be diagnosed [5]. 

The 22q11.2 microduplication syndrome also has variable expressivity and incomplete penetrance, with broad phenotypes, thus hampering the diagnosis [6]. It is worth mentioning that a noticeable inter and intrafamilial variability in the phenotype exists among the individuals carrying this syndrome [7]. Some patients have mild to severe abnormalities, while others can even appear phenotypically normal [8]. Clinical manifestations are characterized by facial dysmorphic features, velopharyngeal insufficiency with or without cleft palate, cardiovascular defects, kidney anomalies, growth delay, and psychiatric disorders [9]. In many cases, the diagnosis of duplication is given when a deletion is suspected, based on the clinical features [10].

Emanuel Syndrome is the most frequent recurrent non-Robertsonian translocation in humans [11]. It has a distinctive phenotype, comprising craniofacial dysmorphism (including preauricular tags or sinuses, micrognathia, ear anomalies, and cleft or high-arched palate), genitourinary abnormalities, congenital heart defects, severe intellectual disability, and delay in developmental milestone [12].

CES is characterized by the existence of a small supernumerary dicentric bisatellited chromosome representing an inv dup(22) (q11.2) [13]. The incidence has been estimated to be approximately 1/50,000–1/150,000 live births [14]. It has a wide spectrum of clinical manifestations, being the three main characteristics of coloboma, anal, and preauricular anomalies. Nonetheless, these clinical manifestations are barely present in 41% of patients, which might indicate that this syndrome is underdiagnosed [15,16]. 

The 22q11.2 region is structurally complex and one of the most unstable regions of the human genome as it contains several specific low-copy repeats (LCRs), designated LCR22A to LCR22H, also known as LCR22-2–LCR22-8, which have a high level of sequence identity in common (more than 95%), predisposing them to non-allelic homologous recombination. This leads to unequal crossovers during meiosis, resulting in deletions, duplications, and inversions [17,18,19]. Hypervariability in the organization of LCR22, especially LCR22-A, is specific to humans, suggesting that non-human primates have less complex haplotypes [20]. Moreover, it is becoming apparent that genetic and epigenetic changes, both within and outside chromosome 22q11.2, can dramatically sway the clinical phenotypes of 22q11.2del [21].

The definitive diagnosis is performed by a genetic study via fluorescence in situ hybridization (FISH), multiplex ligation-dependent probe amplification (MLPA), and chromosome microarray (CMA) [22]. Signs and symptoms and physical examination can guide the diagnosis in some cases, for instance, after detection of specific heart conditions with follow-up or infections early in life, leading to an immune consultant, although the first line of diagnosis is the techniques previously mentioned. As mentioned above, clinical manifestations can be subtle, especially in duplications. Thus, there is a significant possibility of underdiagnosed individuals [23]. Although there has been progress in the diagnostic techniques and treatment of children suffering from 22q11.2 DS in the last years, previous studies have shown that mortality in pediatric patients has a median age of 5 years in which the main cause of death is due to congenital heart disease [24]. In addition, it has been demonstrated that adults have diminished life expectancy and increased risk of sudden death [25]. It has also been shown that they die prematurely in comparison to their unaffected siblings [26]. Limited information is available regarding mortality rates in the remaining 22q11.2 rearrangements. 

Herein, we present data on causes of mortality and average age of death and the existing associated risk factors in 223 patients with 22q11.2 rearrangements. Furthermore, we aimed to establish if there was any significant difference regarding mortality among the different types of rearrangements.

## 2. Patients, Materials, and Methods

### 2.1. Clinical Criteria

Participants for this study were recruited prospectively between 1994 and 2023 from patients referred to the Institute of Medical and Molecular Genetics (INGEMM) of La Paz University Hospital in Madrid, Spain, from neonatology, pediatrics, cardiology, neurology, clinical genetics, and other medical specialties, who had signs and symptoms and/or clinical phenotype compatible with any 22q11.2 rearrangement. Our sample included 223 patients (120 males and 103 females) with confirmed diagnoses through molecular techniques (FISH, MLPA, and CMA). Some patients with mutations in Tbx1, the key gene whose haploinsufficiency is linked to the same congenital defects, may not have been identified. Patients with compatible clinical symptoms but with confirmed negative diagnoses have not been included in this study. In the same way, relatives of patients who have been molecularly confirmed with 22q11.2 rearrangements have also been added to the study, regardless of the presence or absence of symptoms.

Of these 223 individuals, 27 were deceased (10 females and 17 males). We excluded one patient, whose cause of death was not directly related to the 22q11.2 rearrangement. Moreover, in our cohort, there were five fetuses that we did not include in the final cohort because their mothers carried out a voluntary termination of pregnancy. Therefore, the final number of deceased individuals for this analysis was 21.

Information was gathered retrospectively at the time of the inclusion from the patients’ clinical records and prospectively thereafter. To establish the cause of death, we also obtained the patients’ clinical records and postmortem results. Data collection and sampling were performed with prior written informed consent. This study had the approval of the Institutional Board of Research from Hospital Universitario La Paz (CEIC: HULP PI 347). 

### 2.2. Chromosomal and Molecular Studies

All patients have been diagnosed with different molecular techniques (FISH, MLPA, and/or CMA). Karyotyping analyses were conducted on GTG-banded metaphases at an approximate resolution of 550 bands. The analyses followed standard laboratory protocol utilizing Chromosome Kit P (Euroclone; Siziano, PV, Italy). Additionally, FISH analyses were performed using various probes from Kreatech Biotechnology B.V (Amsterdam, The Netherlands) and Vysis Inc. (Downers Grove, IL, USA), according to standard laboratory protocols. 

Different MLPA kits (p023, p250, and p324) were applied to detect microdeletion or microduplication at 22q11.2. All kits were provided by MRC-Holland (Amsterdam, The Netherlands). Data analysis was performed following the manufacturer’s protocols with Coffalysser version 9.4, also from MRC-Holland (Amsterdam, The Netherlands). 

CMA analysis was performed using our custom platform (KaryoArray^®^ v3.0) [27]. The data were analyzed with the Agilent Genomic Workbench software (v.7.0; Agilent Technologies, Santa Clara, CA, USA) using the default CGH analysis method and interpreted by referencing available databases such as DGV, ClinGen, and DECIPHER. All genomic coordinates were represented using GCRh37 (hg19) in UCSC Genome Browser database (https://genome.ucsc.edu/).

### 2.3. Study Limitations

The limitations of this study rely on the partially complete clinical information since the data we gathered from the clinical records were not always entirely complete. This includes a lack of exact timing of certain clinical manifestations, information regarding inheritance, or method and age of death. Furthermore, it is possible that, since some of these patients’ deaths dated back approximately 20 years, at that time, diagnosis techniques were less accurate compared to how they are nowadays. Altogether, this could have led to a possible under-recognition and/or under-diagnosed proportion of individuals with these rearrangements. 

The sample of patients was young, with a median age of 22.54 years. However, when we look at the age range, it was significantly heterogeneous, with the youngest patient being 5 days old and the eldest being 87 years old. Moreover, this study has the particularity that we have included fetuses in our cohort. Similarly, we have decided to include all types of rearrangements, not performing a separate analysis of each one.

## 3. Results

There were 223 individuals included in this study, 103 were female (46.2%) and 120 were male (53.8%). We have collected information from patients who were followed from 1994 to 2023, representing an extensive follow-up and continuous recruitment of individuals. The median age of inclusion in this cohort was 22.54 years [3–87.72]. In our cohort, there were 89 pediatric patients (39.91%) and 129 adults (57.84%). We have also included five fetuses (2.24%) who had a median age of 20 weeks until the termination of pregnancy, with a range between 19 to 23 weeks. Regarding ethnicity, 209 (93.72%) were White Mediterranean (European non-Finnish), 10 (4.48%) were Amerindian (South American), 2 were Arabic (0.89%), 1 was Chinese (0.44%), and 1 was White European (Kazajistan) (0.44%). 

All patients included herein had a confirmed molecular diagnosis achieved through different molecular techniques: 88 with MLPA (39.46%), 65 with FISH (29.14%), 59 with CMA (26.45%), 7 with FISH and MLPA (3.13%), 2 with FISH and CMA (0.89%), and 2 with CMA and MLPA (0.89%). In total, 198 individuals had microdeletion (88.78%), 19 had microduplication (8.52%), 2 had both microdeletion and microduplication (0.89%), 2 had a derivative chromosome 22 (0.89%), 1 individual had both a derivative chromosome plus a microdeletion (0.44%), and 1 patient had a marker chromosome, having been reported as a partial trisomy (0.44%). 

Most rearrangements in the whole cohort have been identified as de novo, being 131 out of 223 (58.74%). In total, 29 were inherited from one parent (13%) and 63 patients had unknown inheritance (28.25%). In patients with deletions, 17 out of the 198 deletions were inherited (8.5%), while 11/19 of the duplications were inherited, representing more than half of the cases (57.89%). One out of the two cases of a derivative chromosome was also inherited, representing half of the patients (50%) as well. 

Regarding mortality of the cohort, we observed 27 deaths (12.1%). However, there was one patient who died at the age of 82 years who never presented with clinical symptoms related to the rearrangement of the 22q11.2 chromosome. Indeed, he was diagnosed after routine genetic testing because an offspring (grandchild) had been diagnosed with this syndrome. His cause of death had no connection with the 22q11.2-related phenotype. Therefore, we have decided not to include him in the total cohort. Similarly, five individuals (18.51%) were fetuses whose parents decided termination of pregnancy (TOP). Consequently, the total number of deceased individuals for further analysis was 21, representing 9.4% of our cohort of 223 patients. Of these 21, 8 were female (38%) while 13 were male (62%). The median age of death was 3 months and 18 days (ranging from 3 days to 34 years). There were 17 patients who died at pediatric age (80.95%), 3 who died at an adult age (14.28%); in 1 patient (4.76%), the age of death was unknown. Regarding their molecular defects, rearrangements included 19 microdeletions (90.47%), 1 microduplication (4.76%), and 1 patient with a marker chromosome (4.76%). In total, 18 patients were White Mediterranean (European non-finish) (85.71%) whereas 3 were Amerindian (South American) (14.28%). 

Based on the clinical information of the patients, the median age at diagnosis was 6 days, with a wide range between 1 day and 20 years. Twenty patients were diagnosed at the pediatric age (95.23%). From them, 7 out of 20 (35%) were diagnosed within the first day of life. These patients had severe congenital heart disease (CHD) plus other multisystem symptoms, which could have led to the early diagnosis of the syndrome. Only one patient was diagnosed at an adult age, at 20 years (4.76%). 

### 3.1. Causes of Death and Clinical Findings

Table 1 lists the causes of death according to the patients’ clinical information. Cardiovascular causes account for the majority of deaths. It was registered as a single cause of death in 12 patients (57.1%) or in combination with other causes in 3 patients (14.28%) (cardiovascular added to respiratory failure, cardiovascular plus hematological causes, and cardiac added to sepsis). Considering both groups together, mortality from cardiovascular causes accounts for up to 71.42%. The second most frequent cause of death was sepsis in two patients (9.52%). One patient died from respiratory failure (4.76%) and one from renal failure (4.76%). Finally, information regarding the cause of death was unavailable in two patients (9.52%).

Additional patient information, clinical findings, molecular diagnosis, and other details on the cause of death are provided in Table 2. As mentioned above, most patients died due to cardiovascular causes. Specifically, five died as a result of cardiorespiratory arrest secondary to cardiac surgery, four died due to complications derived from their CHD, one died of ischemic heart disease, one due to sudden death, and one as a result of a cardiorespiratory arrest not related to cardiac surgery. In addition, in combination with other causes, one patient’s cause of death was CHD and bleeding due to thrombocytopenia, one cardiorespiratory arrest plus sepsis, and one because of hypoxemia and progressive hemodynamic failure after cardiovascular surgery. An interrupted aortic arch (IAA) is the most common cardiovascular defect in patients with 22q11.2 rearrangements, followed by truncus arteriosus (TA) and tetralogy of Fallot (TF) in the third place [28]. Yet, in our cohort, the most frequent cardiac defect was TA, present in 7/21 patients, representing 33.33%, and then IAA present in 6/21 patients, representing 28.57%. Regarding TF, similarly to what was established in previous articles, it is in third place in order of frequency, having 3/21 patients with this condition, representing 14.28%. In addition, four individuals had neuropsychiatric symptoms (19.04%) and two patients also had epilepsy (9.5%). Additional signs and symptoms were identified in 18 out of the 21 patients (85.71%), while 3 individuals presented with CHD alone (14.28%). The most frequent phenotypic additional defects included dysmorphic features and it was present in 13 patients (68.42%). Other features such as gastrointestinal problems and thyroid anomalies were present in five patients (27.78%) and four individuals (22.22%), respectively. The patient with marker chromosome presented with clinical features resembling CES (MIM# 115470), including dysmorphic face, aplasia of both thumbs and radius, lumbar hemivertebrae, anal atresia, cholestasis, and cleft palate.

In total, 13 out of 21 patients underwent cardiac surgery (61.9%). Of those 13 individuals, 2 died during surgery (15.38%), 5 died in the immediate postoperative period (38.46%), 5 died some days after surgery (38.46%), and in 1 patient, this information was not available. Of the five patients who died after surgery, survival rates were variable, with a range between 1 year and 27 years (median age of 15 years). Nine patients had surgery before 45 days of life (69.23%) and seven within the first month of life. It should be noted that the patient deceased at 27 years old died due to a complication of a hemodynamic catheterization procedure. The individual who had not undergone cardiac surgery was the only one who died later, at 34 years old. 

**Figure 1:**
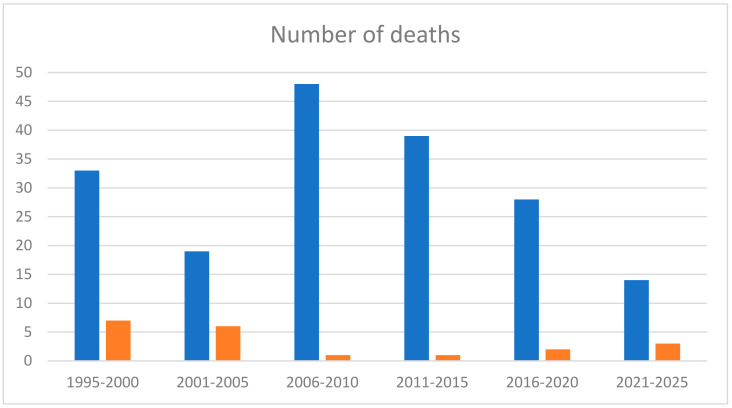
Mortality divided every 5 years based on the number of deceased patients and the number of patients with new diagnoses. Blue bars denote the number of diagnoses and orange bars the number of deceased patients.

Figure 1 shows mortality divided every 5 years based on the number of deceased patients and the number of patients with new diagnoses in the same period of time. Most deaths in children are concentrated in the decade between 1995–2005 whereas the vast majority of diagnoses were conducted between 2006 and 2016.

To the best of our knowledge, all deceased patients were receiving medical care and were followed periodically by medical specialists in tertiary hospitals. In addition, they were taking prescribed medications and none were using recreational drugs or smoking cigarettes.

### 3.2. Genomic Findings

Among the 21 deceased individuals, 16 were diagnosed with FISH (76.19%), 2 with CMA (12.5%), 2 with MLPA (12.5%), and 1 with CMA and MLPA (4.76%). In total, 19 individuals (84.21%) presented microdeletions, 17 showed the typical A-D while 1 carried the nested 1.5 Mb A-B deletion (5.26%) and there was 1 individual with no information (5.26%) on the size of the deletion. One patient carried a microduplication of 1.5 Mb (4.76%) and one carried a marker chromosome 22 (4.76%).

Of the 19 patients with microdeletions, 2 individuals inherited the microdeletion from their mothers (10.52%), which was diagnosed in adulthood at the time of their child’s diagnosis. However, both had psychiatric symptoms, while one additionally presented short stature and dysmorphic faces. The remaining 17 individuals had a de novo rearrangement (89.47%). Notably, the 1.5 Mb A-B microdeletion (individual 1) was inherited, consistent with the previous literature, which reports that nested 1.5 Mb rearrangements tend to have a familial aggregation [29]. 

In total, 3 out of 21 patients (14.28%) presented additional genetic rearrangements. One patient (ID 4) carried a de novo microdeletion at 22q12.3q13.1 of 3.5 Mb as well as a de novo microduplication at 22q13.31 of 2.53 Mb, both described in the Genomic Variants Detection, and could explain the severe hearing loss present in this patient. Another individual (ID 5) carried a pericentric inversion at chromosome 9 (inv(9)(p11-q13), inherited from his father, which is a morphological variant with no impact on the phenotype. Finally, a further patient (ID 20) had a balanced translocation t(14;16)(q32.3;p13.1) inherited from his asymptomatic mother

## 4. Discussion

### 4.1. Mortality of 22 q11.2 Deletions Is Higher than in Other Rearrangements

Probably due to the severity of the heart diseases, the mortality of 22 q11.2 deletions is higher than in other rearrangements but we cannot conclude whether 22q11.2 rearrangements themselves represent an independent risk factor for mortality after cardiac surgery. Patients with microdeletions are more symptomatic and usually present additional clinical features. It is well known that 22q11.2 individuals are associated with an increased risk of recurrent infections [30]. Three cases from our cohort died because of severe sepsis and only one presented lymphopenia with low T lymphocytes CD4+. Also, the contribution of non-immunological factors, such as palatal abnormalities, bronchomalacia, gastroesophageal reflux, asthma, and rhinitis seems to be associated with recurrent infections [31]. Previous series revealed a higher frequency of sepsis and/or infections, along with longer administration of antibiotics, in the postoperative course of patients with 22q11.2 rearrangements [26,32,33,34]. We observed several patients with post-surgical infections. In fact, one patient (ID 15) who underwent a Nissen fundoplication for long-standing gastroesophageal reflux disease developed sepsis due to *Pseudomonas* spp. 10 days after surgery and 12 days later passed away due to a fatal *Candida* spp. sepsis. 

Of the 21 deceased individuals, only four had neuropsychiatric symptoms; three adults and a child who died at 15 years old, all of them presenting with 22q11.2 microdeletion. Psychiatric disorders are more commonly seen during adulthood compared to childhood [35]. However, we have not seen a correlation between neuropsychiatric features and mortality among our cohort. 

Of the total deaths, the vast majority corresponded to patients with microdeletions, only one patient to a derivative chromosome 22 and one due to a duplication. The latter also had an additional distal duplication at 22q13.31 and a deletion in 22q12.3q13.1. To date, there is no evidence linking mortality to other types of 22q rearrangements beyond the microdeletion syndrome. Microduplication syndrome has variable expressivity, with some patients even appearing phenotypically normal and with a lower prevalence of CHD in comparison with the microdeletion syndrome, which could influence the lower incidence of death [7,36]. Patient 14 was diagnosed with a partial trisomy (47,XY,+mar(22).ish 22q11.2(D22S75X2)). He died from a cardiorespiratory arrest and presented with many features associated with CES, including dysmorphic faces, aplasia of both thumbs and radius, lumbar hemivertebra, anal atresia, and cleft palate. This patient was diagnosed in 1998, when diagnosis techniques were less specific. Thus, instead of having a marker chromosome, it is most likely that he had a derivative chromosome and his diagnosis CES. Nowadays, there is scant information about survival in patients with CES. Nonetheless, it is known that patients generally have a good prognosis and mortality is usually due to complications derived from the malformations itself, such as cardiac failure, bronchopneumonia, sepsis, liver failure, or biliary atresia [37]. 

### 4.2. Congenital Heart Diseases Are the Determining Factor of Mortality

Overall, the most frequent cause of death was cardiovascular in nature, either as a single cause of death or in combination with other factors, representing 71.42% of deaths [26,38]. Since all deceased patients presented with CHD, it represents one of the most important factors associated with decreased survival, as reported before [26]. The vast majority had severe heart defects, mainly interrupted aortic arch, TOF, and pulmonary valve atresia, among others. Thus, right ventricular failure is an important cause of morbidity and mortality in patients with 22q11.2 syndrome, either before or after cardiac surgery [39], which is also related to pulmonary arterial hypertension or aortopulmonary collaterals, leading to a worse prognosis [40,41]. The earlier these patients were diagnosed, the more severe the cases were; this is due to the complex phenotype. There are several studies focusing on life expectancy in patients with CHD. Perez-Lézcure Picarzo et al. analyzed the mortality of CHD in Spain over 10 years in children under one year of age [32]; similarly to our data, most of them died during the first week of life. The proportion of deaths was 4.58%, lower compared to our study, which could indicate the influence of the extracardiac conditions present in patients with chromosome 22q11.2 rearrangements on mortality and reduced survival in children. Another study carried out in Sweden reported improved survival in children with CHD since 1980 with a decline in mortality to less than 20% [42]. Regarding adults with CHD, it has been reported that they have a lower survival than patients without this condition [43]. A systematic review and meta-analysis concluded that the mortality rate is higher during the first year of life; however, survival decreases progressively after infancy and into adulthood [44]. A further series reported that the median survival for an adult of 18 years with severe CHD was 53.3 years and in the case of mild CHD was 75.4 years [45]. All this evidence shows that adults with CHD may have a shorter life expectancy. In our cohort, patients died much earlier compared to previous studies [25,26], suggesting that other factors may interfere with mortality, not only due to cardiac causes but also extracardiac, having a multifactorial nature [25]. Thus, three adult patients died from sudden death, cardiorespiratory arrest secondary to a surgical complication of a cardiac catheterization, and cardiorespiratory arrest secondary to a septic shock.

Thus, deaths in the neonatal period depended on the severity of the heart disease. In our cohort, the most frequent cause of death observed was cardiorespiratory arrest secondary to cardiac surgery. In total, 13 out of the 21 patients underwent surgery and two did not by their self-decision. It is important to note that more than half of the patients underwent surgery when they were less than a month old. Newborns have a low body weight, demand advanced surgical skills, and have a higher risk of anesthesia, consequently, surgery is more difficult in this period [46]. Furthermore, patients with genetic syndromes have a risk of prematurity and low birth weight, affecting recovery after surgery and decreasing early and immediate survival [47]. Moreover, as heart defects are particularly complex, they require surgical reinterventions, hampering the prognosis [47,48,49]; there were even six patients who underwent various reinterventions and three who finally died due to complications derived from it. 

There are certain cardiovascular anomalies that are associated with a worse prognosis. Kyburtz et al. identified pulmonary vessels’ anatomy as an important risk factor for mortality and reintervention in patients undergoing repair surgery [49]. In addition, Mahle et al. suggested that patients with del22q11.2 have a higher mortality rate in comparison with healthy individuals due to the presence of more severe artery hypoplasia in patients with pulmonary atresia (PA), ventricular septal defect (VSD), and major aortopulmonary collateral arteries (MAPCAs) [50]. Carotti et al. also demonstrated that del22q11.2 is significantly associated with mortality among patients who underwent surgery for such anomalies [51]. In contrast, Michielon et al. concluded that del22q11 does not represent risk factors for mortality after repair of conotruncal anomalies, though the authors excluded in their study individuals with PA, VSD, and MAPCAs [41]. 

O’Bryne et al. analyzed postoperative outcomes in patients who underwent cardiac surgery correction of interrupted aortic arch (IAA) and truncus arteriosus (TA) and did not see higher mortality in patients with del22q11.2, stating that increased mortality was because of the abnormal anatomy and not due to the deletion itself [48]. Three other studies were not able to detect any increased risk of mortality in patients with del22q11.2 after cardiac surgery [40,47,52]. In our cohort, all deceased patients had complex CHD, including IAA, pulmonary hypertension (PAH), and pulmonary valve atresia. Moreover, we have seen several patients with kidney failure who required dialysis and one patient who died from postsurgical kidney failure. In addition, all patients had additional comorbidities, including hydroelectrolyte disturbances, hypocalcemia, and infections, worsening the postsurgical prognosis [33,38]. As expected, a study demonstrated more postoperative complications after cardiac surgery in the group of patients with genetic abnormalities [43]. It is important to mention that five of the patients who underwent cardiac surgery had arrhythmias in the postoperative period, leading to death in three of them.

Regarding fetal deaths, although we did not take them into account in our analysis, we consider it relevant to provide some statistical information. A previous study reported an incidence of ~1/1500 of fetuses with 22q11.2 rearrangements when analyzing miscarriage samples [53]. In our cohort, we observed five fetal deaths out of the total number of deaths, which corresponds to 19.23%. From the information we were able to collect, two had cardiac abnormalities reported on ultrasound. These data are important given that the main cause of death is cardiac defects [3]. Furthermore, three fetuses had the deletion inherited from the father. This information is also important given that it denotes the importance of analyzing the parents to evaluate the reproductive risk in the future, considering that approximately 7% of parents who have a child with the deletion also have the disease [3].

### 4.3. Decrease in Mortality in the Past Decades Depends on the Improvement in Surgical Techniques for Heart Disease

Due to the complexity of cardiac defects, most patients underwent palliative surgery instead of reparative surgery. In our study, the majority of the patients have been surgically intervened on in the late 90s and early 2000s. Over the last few decades, there have been advances in cardiac surgeries, which led to an increased survival rate of these patients [33,54]. In fact, as shown in Figure 1, most deaths occurred in the decade from 1995 to 2005, with all cases being pediatric and the most frequent cause of death was related to cardiac surgery.

### 4.4. Comparison with Other Series

22q11.2 genomic rearrangements are a significant cause of morbidity and mortality of individuals, with high death rates (≈12.46%) [25,26,38,55]. In our large cohort of patients, we found that 9.4% of individuals died, being in approximately 1/11 individuals, which is slightly less in comparison with previous reports [25,26,38,55]. Based on age groups, the mortality rate in pediatric patients was 19.54%, with most deaths occurring during the first year of life, with a median of 2 months. These results suggest a higher mortality rate in toddlers compared to previous studies [38]. Regarding adult cases, only 3 out of the 129 adults died, representing 2.32%, with a median age of 27 years. These results show a lower mortality rate in adults compared to previously published series but at an earlier age [25,26]. This lower survival could be explained by the fact that our cohort was relatively young, with a median of 22.54 years, and a huge number of children. 

## 5. Conclusions

This study adds information on the causes of mortality in patients carrying 22q11.2 rearrangements. Genetic and molecular tests must be performed forthwith in order to have an early diagnosis and thus be able to provide a multidisciplinary approach to the patient, with the corresponding genetic counseling and long-term follow-up. Individuals carrying these rearrangements are at risk of premature death, in both children and adults. Although complex heart defects are significantly related to mortality, other findings may also contribute to mortality, leading to a multifactorial etiology. Individualized surgical approaches are needed according to the underlying cardiac defect in each patient, as well as careful post-surgical management, necessary to avoid short- and long-term complications. Further studies and prospective follow-up of further patients are necessary to improve our understanding of mortality causes, as well as to assess survival and predict risk factors in patients with these rearrangements, especially regarding microduplications and derived and marker chromosomes.

## Figures and Tables

**Table 1:** Causes of death according to clinical records. Five fetuses with TOP are not included in the table.

Cause	Age at Death: Range in Years (Median)	Number of Patients	Percentage (%)
Cardiac	1 month, 15 days (range 3 days–27 years)	12	57.14
Sepsis	1 year, 7 months (range 15 months–2 years, 11 months)	2	9.52
Unknown	-	2	9.52
Cardiac + respiratory failure	15 days	1	4.76
Cardiac + hematological	15 years	1	4.76
Respiratory failure	1 year	1	4.76
Renal failure	33 days	1	4.76
Cardiac + sepsis	34 years	1	4.76
Total	3 months, 18 days (range 3 days–34 years)	21	100

**Table 2:** Main clinical features and molecular characteristics of deceased patients.

Patient Information			Details of Death			Clinical Findings			Molecular Genetic			Comments
Case	Gendre	Ethnicity	Age at Diagnosis	Age of Death	Cause of Death	Cardiac	Neuropsychiatric	Others	Rearrangement	Inheritance	Test Platform	
1	F	White Mediterranean (non-Finish)	7 years, 3 months, 7 days	27 years	Cardiorespiratory arrest secondary to cardiac surgery	TA type II (surgically intervened 3 months), VSD, pulmonary stenosis, severe cyanotic CHD, RAA. Surgically intervined.	Hyperactive behavior, epilepsy, ADHD, GDD	Hypoparathyroidism, microcephaly, dysmorphic facies, myopia (8 dioptres)	Deletion A-B	Inherited mother	FISH	
2	F	White Mediterranean (non-Finish)	7 days	Unknown	Unknown	TF, pulmonary valve agenesis.			Deletion	De novo	FISH	
3	M	White Mediterranean (non-Finish)	1 day	5 days	Ischemic heart disease	TA type 1, anomalous left subclavian artery, VSD.		SGA, dysmorphic facies, slightly elongated fingers	Deletion A-D 2.56 Mb	De novo	CMA	Ultrasound week 31 with suspicion of structural congenital heart disease. Has a phenotypically normal identical twin brother
4	M	Amerindian (South-American)	4 days	15 years	CHD and bleeding due to thrombocytopenia	LV hypoplasia, DORV, single atrium, mild tricuspid insufficiency, CoA, atrioventricular conduction disorders. Surgically intervined.	GDD	Severe hearing loss, brachycephaly, dysmorphic features, aphonia, visual deficit, ischemic colitis, biliary sludge, portal hypertension secondary to Fontan surgery.	Duplication 22q11.23-q12.1 (1.55 Mb)	De novo	CMA + MLPA	Ultrasound week 25 with suspicion of structural congenital heart disease. Deletion 53.7 Kb in chromosome 6. Deletion 22q12.3q13.1 (3.5 Mb). Duplication 22q13.31 (2.53 Mb)
5	M	White Mediterranean (non-Finish)	1 day	15 days	Hypoxemia and progressive hemodynamic failure after cardiovascular surgery	TA type II, isolated aortic arch, PAH, RAA. Surgically intervined.		Dysmorphic facies, palate with abnormal dermatoglyphsv Velopharyngeal incompetence, microcephaly, bilateral skin ectasia, small umbilical hernia	Deletion A-D	De novo	FISH	Carrier of an inversion on chromosome 9 [46, XY, inv (9) (p11q13)] inherited from his father. Benign variant.
6	M	White Mediterranean (non-Finish)	2 years	2 years, 11 months	Sepsis	VSD, pulmonary valve atresia, RAA, $patent DA.			Deletion A-D	Unknown	FISH	
7	M	White Mediterranean (non-Finish)	1 day	9 days	Cardiorespiratory arrest secondary to cardiac surgery	IAA type A, DA, AVVR, single atrium, LSVC draining the coronary sinus, CAVC. Surgically intervined.		Multifactorial anaemia.	Deletion A-D	Unknown	FISH	Ultrasound week 30 with diagnosis of CHD+single umbilical artery
8	F	White Mediterranean (non-Finish)	6 days	10 days	Complications derived from CHD	TA, VSD, PAH.		Hypocalcemia	Deletion A-D	Inherited mother	FISH	
9	F	White Mediterranean (non-Finish)	26 days	33 days	Post-surgical renal failure	VSD, TA type I, extrasystoles, PAH. Surgically intervined.		Dysmorphic facies	Deletion A-D	De novo	FISH	
10	M	Ameridian (South American)	1 day	1 year	Respiratory failure ventilatory restrictive with hypercapnia	Pulmonary atresia, VSD, RAA, pulmonary hypoaflow, pacemaker, arterial hypertension, MAPCA. Surgically intervined.		Chronic respiratory disease, CMPA, GERD, dysphagia	Deletion A-D (2.61 Mb)	De novo	CMA	Ultrasound week 30 with diagnosis of CHD
11	F	White Mediterranean (non-Finish)	1 month, 8 days	21 years	Sudden Death	IAA, VSD, ASD. Surgically intervened.	ASD, GDD, seizures, attention deficit	Premature, short stature, arched palate, thyroid nodules, dysmorphic facies, altered phosphocalcium metabolism	Deletion A-D	De novo	FISH	
12	F	White Mediterranean (non-Finish)	1 day	2 months, 13 days	Cardiorespiratory arrest secondary to cardiac surgery	TF, pulmonary valve agenesis with severe pulmonary stenosis, RAA, severe RV dilation, VSD. Surgically intervined.		Symptomatic hypocalcemia, hypothyroidism, SGD, multifactorial anemia, polymalformative syndrome, ischemia in left leg, dysmorphic features	Deletion A-D	De novo	FISH	
13	M	White Mediterranean (non-Finish)	16 days	26 days	Complications derived from CHD	IAA type B, large VSD, bicuspid aortic valve, subaortic stenosis, large patent DA, ascending aorta hypoplasia, ASD. Surgically intervined.		Dysmorphic facies, hepatopathy with jaundice and coagulation disorders, hypocalcemia, lymphopenia	Deletion A-D	De novo	FISH	
14	M	White Mediterranean (non-Finish)	10 days	2 months, 2 days	Cardiorespiratory arrest	VSD, ASD, small DA, anomalous pulmonary venous drainage. Surgically intervined.		Premature, dysmorphic facies, aplasia of both thumbs and radius, lumbar hemivertebra, anal atresia, cholestasis, cleft palate	Marker chromosome 22 (partial trisomy)	De novo	FISH	Cat Eye Syndrome
15	M	White Mediterranean (non-Finish)	5 days	17 months	Sepsis	VSD, IAA, auriculoventricular block with external pacemaker. Surgically intervined.		Lymphopenia with low CD4, coloboma, bronchopulmonary dysplasia, GERD, hiatus hernia, multiple thromboses, velopharyngeal incompetence	Deletion A-D	De novo	FISH	
16	F	White Mediterranean (non-Finish)	10 days	8 months	Unknown	TA type I.			Deletion A-D	De novo	FISH	
17	F	Ameridian (South American)	1 month, 20 days	4 months, 25 days	Complications derived from CHD	VSD, pulmonary valve atresia.		Dysmorphia	Deletion A-D (3 Mb)	De novo	MLPA	
18	M	White Mediterranean (non-Finish)	1 day	3 days	Cardiorespiratory arrest secondary to cardiac surgery	Severe aortic stenosis, IAA type B, VSD, bicuspid aortic, valve, DA, patent foramen ovale. Surgically intervined.		Dysmorphic features	Deletion A-D	De novo	FISH	
19	M	White Mediterranean (non-Finish)	20 years, 21 days	34 years	Cardiorrespiratory arrest + sepsis	Pulmonary valve atresia, PAH, VSD, MAPCA. Not surgically intervined.	Learning disabilities, GDD	Dysmorphic features, partial agenesis right hemithyroid, articulation disorder, right severe hypoacusis, polyglobulia, antiphospholipid syndrome, hyperuricemia and gout, mild hypocalcemia, ictus, clubfoot	Deletion A-D (3 Mb)	De novo	MLPA	
20	M	White Mediterranean (non-Finish)	1 day	12 days	Cardiorespiratory arrest secondary to cardiac surgery	TA type I, IAA, mild tricuspid insufficiency, PAH, VSD, ASD ostium secundum, paroxysmal supraventricular tachycardia. Surgically intervined.		Hypoplasia of the depressor labii inferioris muscle, right cryptorchidism	Deletion A-D	De novo	FISH	Translocation t (14;16)(q32.3;p13.1) inherited from the mother
21	M	White Mediterranean (non-Finish)	3 days	7 months	Complications derived from CHD	TF, incompetent persistent foramen ovale, mild PAH.		Dysmorphic features, polymalformation, supernumerary spleen, corpus callosum agenesis	Deletion A-D (3 Mb)	De novo	FISH	

TA: truncus arteriosus. VSD: ventricular septal defect. CHD: congenital heart disease. ADHD: attention deficit disorder. TF: Tetralogy of Fallot. SGD: small for gestational age. LV: left ventricle. DORV: Double outlet right ventricle. CoA: coarctation of the aorta. PAH: pulmonary hypertension. RAA: right aortic arch. DA: ductus arteriosus. AVVR: Atrioventricular valve regurgitation RV: right ventricle. ASD: atrium septal defect. CAVC: complete atrioventricular canal defect. IAA: interrupted aortic arch. LSVD: left superior vena cava. MAPCA: major aortopulmonary collateral arteries. CMPA: cow’s milk protein allergy. GERD: gastroesophageal reflux disease. GDD: global developmental delay. ASD: Autism Spectrum Disorder. Fetuses are not included in the table.

## Data Availability

Data is available for research use only under request.
